# Atlanta Academy of Medicine

**Published:** 1875-08

**Authors:** Robert Battey

**Affiliations:** Reporter


### Atlanta Academy of Medicine.

ROBERT BATTEY, M.D., REPORTER.

ATLANTA, 15th June, 1875.

The President, Dr. John M. Johnson, in the chair.

Dr. Woodhull read notes of some recent cases of dysentery treated with the non-emetic use of ipecacuanha—showing prompt controlling influence of the remedy over the disease. These cases also go to show the facility of the retention of the remedy in large doses without vomiting.

Dr. Taliaferro stated that he had been recently using the method in dysentery and severe forms of diarrhœa with much satisfaction and with no nausea or vomiting. The cures are very prompt and satisfactory. He is delighted with the results thus ar obtained.

Dr. Logan asked whether the occurrence of vomiting interfered with the salutary effects of the remedy.

Dr. Taliaferro had found the relief secured notwithstanding the vomiting in some cases. Finds the results not so prompt and marked in diarrhœa as in dysentery; the former is controlled, but less promptly.

Dr. Woodhull had not found the occurrence of vomiting to interfere with the salutary effects of the remedy in dysentery. Vomiting is less apt to occur in dysentery than in diarrhœa. He does not allow his patients water within four hours after taking the dose.

Dr. Conally reported case of tight prepuce, which when slit up revealed a granular condition of the mucous membrane covering the prepuce and the glans penis. The patient is of scrofulous habit. Asks advice as to treatment, as the parts do not incline to heal.

On call, Dr. Battey suggested the use of alterative doses of the potassic iodide and corrosive sublimate, with the local use of a freshly prepared simple cerate.

Adjourned.

---

ATLANTA, June 22, 1875.

The President, Dr. John M. Johnson, in the chair.

Dr. J. T. Johnson reported that he has under treatment a well marked case of typhoid fever, with distinct enteric lesions.

Dr. W. F. Westmoreland reported case of a young lady of 28. At the age of 15 she was troubled with uterine hemorrhage. At the age of 18 he removed from her uterus a fibrous polypus as large as a hen's egg. On yesterday he removed from her another similar polypus of half the size of an egg. Desires to know if this recurrence of polypus is usual.

Dr. Taliaferro expressed the opinion that a uterine polypus seldom returns, but we may have numerous polypi in the same uterus.

Dr. John M. Johnson had in his care years ago a case of uterine polypus. He treated the case first by a douche of equal parts of tincture of iron and water, and afterwards by applications of the muriated tincture, painted upon the tumor through the speculum. He had the satisfaction to find the polypus soon break down so that he easily removed it. He thinks the tincture of iron very efficacious in endometritis, membranous dysmenorrhœa, mucous polypus, and all forms of leucorrhœa. He has also used it successfully in polypi of the nose and ear.

Dr. Westmoreland asked if Dr. Johnson would treat a uterine fibroid by tincture of iron exclusively.

Dr. Johnson replied yes.

Dr. Westmoreland thought that a true fibrous polypus would not yield to the remedy, whilst the ecraseur would remedy the disease in ten minutes. He admits that the small mucous polypus would yield to the tincture of iron, but he once found the manipulation required in a case to establish the diagnosis of the mucous polypus, ended in the detachment of the growth without any instrument at all, and without bleeding.

Dr. Johnson does not insist upon the fibrous growths, but is certain that he has removed three polypi by the remedy.

Dr. Calhoun stated, in reply to a question, that all the polypi of the ear which he had encountered were fibrous.

Dr. Westmoreland stated that in the nose we have chiefly the mucous polypus—sometimes fibrous.

Dr. Taliaferro stated that the uterine polypus was more frequently mucous, but occasionally fibrous, and an outgrowth from the parynchyma of the uterus itself.

Dr. Westmoreland thought the fibrous polypus of the uterus more common than stated by Dr. Taliaferro.

Dr. Johnson related a case of reduction of paraphymosis by manipulation and without operation. He compresses the glans strongly and afterwards draws the prepuce forwards.

Dr. J. T. Johnson remarked that the method given was generally accepted by the profession at the present day. He suggested that sometimes a little patience in the compression of the glans was necessary to success.

Dr. Taliaferro reported a case of alarming effects in the administration of Squibbs' ether. The respiration ceased; was re-established, and again ceased upon the renewal of the ether. Adjourned.

---

Atlanta, 29th June, 1875.

The President, Dr. John M. Johnson in the chair.

Dr. J. T. Johnson reported the death of the case of typhoid fever alluded to at the last meeting. No autopsy could be obtained.

Dr. Armstrong reported case of cholera morbus in a boy of twelve, with much collapse. There was great cramping of the extremities. On rubbing the limbs to bring on reaction, he observed great tenderness in one of his limbs. He had lately had an attack of fever accompanied by much soreness of the muscles. This afternoon there is extreme tenderness of the muscles of one leg, which rested upon a pillow, and could not be touched excepting with extreme complaint of pain. The limb is a little puffed, but there is no heat, nor redness, nor other symptoms of inflammation. The tenderness does not involve the joints, which can be moved without pain. It is not hyperæsthesia of the skin. The tenderness is confined to the muscles of one leg only. It is, clearly, not rheumatism. What is it?

Dr. Boring saw the case with Dr. Armstrong, and is wholly at a loss for a diagnosis. There is no tenderness in the thigh and none in the ankle or foot. It is not phlebitis. What is it?

Dr. Armstrong, in reply to question, said he was sure there is no pus in the leg. Occasionally the boy has pain in the muscles of the leg when at rest, but ordinarily none unless when touched.

Dr. Baird suggested that the condition is likely that of myalgia, and he thinks galvanism is the remedy.

Dr. Armstrong coincided in the opinions of Dr. Baird as to diagnosis and treatment.

Dr. Baird suggested the possible existence of worms.

Dr. Armstrong replied that the boy had a powerful vermifuge treatment a month ago without effect.

Dr. Todd suggested muscular rheumatism, and thought that alkalies, quinine, and spinal counter-irritation would relieve the case, rather than galvanism. He thinks that rheumatism is a nervous affection.

Dr. Baird reported upon a specimen of dysmenorrhœal membrane submitted to him by Dr. Battey, and exhibited some mounted specimens under the microscope from the membrane. Dr. Baird said: The dysmenorrhœal membrane sent to him for microscopical examination presented the following characteristics: It was about one inch and a half in length, and about three-quarters of an inch in width at its greatest diameter. The specimen was torn across about midway between the cervix and fundus. It represented accurately the inner surface of the uterus; the cervix, the body, the fundus and commencement of the fallopian tubes being as accurately reproduced, with all of their inequalities of surface, as they would have been by a plaster cast.

The interior of the closed membrane was smoother and presented a somewhat glossy or glazed appearance. The structure grew more and more dense as the inner surface was approached. The membrane measured from a line to a line and a half in thickness. The microscope, proved its structure to be cells, simply abundantly proliferated epithelial cells. In the examination he had the valuable assistance of Drs. Rauschenberg and Simpson, and they were unable to detect the presence of connective tissue, or connective tissue cells, fibrous tissue or blood-blessels, and they unite in the opinion that the membrane consists exclusively of proliferated epithelial cells, from the surface of the uterine mucous membrane, in various stages of development.

Dr. Rauschenberg thought the dysmenorrhœal membrane is essentially different from the croupous membrane. The latter is decidedly pathological, the dysmenorrhœal membrane, on the other hand, is histological in character. The tissue in the latter is physiological tissue, whilst the croupous membrane is wholly and essentially pathological tissue.

Dr. Battey read a volunteer essay upon membranous dysmenorrhœa. (See page 257.)

Dr. Taliaferro remarked that some recent authorities claim that the mucous lining of the uterus is shed always at the menstrual period, but always in a disintegrated form when the menses are healthy. It is also claimed that in membranous dysmenorrhœa the membrane is simply thrown off entire, and not disintegrated. Others assert that only the epithelium is lost either in healthy menstruation or in dysmenorrhœa. He coincides in opinion with the latter authorities.

Dr. Woodhull asked if the usual treatment of the disease had been entirely local, or has constitutional treatment been relied upon at all for the cure.

Dr. Battey replied that every remedy which promised good, either locally or constitutionally, had been fully tried.

Dr. Woodhull suggested that the muriate of ammonia, if persisted in for a long time, and the system well saturated with it, might prove a hopeful remedy.

Dr. Battey replied that the muriate of ammonia was one of the chief constitutional remedies relied upon, but without avail.

Dr. Battey read a communication from Dr. C. H. Harris, of Polk county, (see page 262) and desired the members to experiment with the new suggestion of Dr. Harris in suitable cases.

Dr. Battey also exhibited for Dr. L. Alexander, of Yorkville, S. C., a modification of Albert Smith's modification of Hodge's bow pessary, and read from the letter of Dr. Alexander explanatory of the instrument and its use:

"You recognize the addition of an air bag to the Albert Smith closed lever, which combination has given me more satisfaction than any other instrument I have used; particularly in cases of long standing; and I think, upon trial, that some of the objections generally found in other instruments of the kind are, in a measure, overcome. I think that unless the uterus is quite low in the vagina the lever alone falls short of the object desired; and should it not be properly adjusted there is danger of producing retroflexion in trying to overcome retroversion. With this combination a smaller pessary answers, besides giving the womb a soft and elastic cushion to rest upon, or against, in the descent of the abdominal viscera. It is only required to place the patient in the knee-and-breast position, insert the instrument, with the bag collapsed and thrown back; then inflate the rubber bag to sufficient size with a Mattson's syringe, and the bag by its distension, will replace the uterus. I am sure that upon regaining the erect posture a vaginal examination will show the organ in its proper place. The bag may be distended to any size, though it is only necessary to inflate enough to cause it to adapt itself to the adjacent parts. The tube can be cut off below when it is tied, and afterwards reunited by simply slipping a piece of fine tube into the cut ends, and in that way bring them together again. Tieman & Co., New York, make the instrument, and inform me that the wholesale price will be $2,25 each."

In the application of Alexander's pessary, the elastic bag, which is turned backwards in the introduction, when inflated executes a lever movement in the required direction, as illustrated to the Academy, which seems to be admirably calculated to reposit the fundus in retroversions and retroflexions of the uterus. He will give the new instrument a trial, when a suitable case offers, and report the result.

Academy adjourned to the first Tuesday in September next.

